# Study on Factors Affecting Estrus Synchronization in Smallholder Dairy Farming Systems of Tigray, Northern Ethiopia

**DOI:** 10.1155/2022/2435696

**Published:** 2022-04-27

**Authors:** Dagmawit Kibre, Gebregiorgis Ashebir, Berihu Gebrekidan, Haben Fesseha

**Affiliations:** ^1^College of Veterinary Science, Mekelle University, Mekelle, Ethiopia; ^2^School of Veterinary Medicine, Wolaita Sodo University, Wolaita Sodo, Ethiopia

## Abstract

**Background:**

Reproductive biotechnology, such as estrus synchronization, can quickly boost the genetics of local cattle breeds, shorten generational cycles, and spread genetic material within populations of breeding animals.

**Methods:**

A cross-sectional study was performed on 154 purposively selected smallholder dairy owners using a semistructured questionnaire to assess factors that influence the success of estrus synchronization in smallholder dairy farms located in Agula, Wukro, and Enderta districts, Northern Ethiopia.

**Results:**

The estrus synchronization programme was positively accepted by 39.6% of illiterate participants. However, the education level and marital status of the participants had no significant association (*p* > 0.05) between the different study sites. On the other hand, approximately 10% of interviewees did not have awareness of dairy cattle estrus synchronization technology at the time of implementation, whereas 36 (23.4%) farmers who were aware of estrus synchronization gave negative feedback on the technology. Factors such as breed, management system, feed type, feeding, and watering frequency significantly varied (*p* < 0.05) among the three study sites, whereas the breeding practice had no significant association (*p* > 0.05) within these districts. The cause of failure during AI, awareness about synchronization, satisfaction with the AI, and estrus synchronization service have a significant variation (*p* < 0.05) within the three districts. On the other hand, the cause of AI failure, awareness about AI programs, source of synchronization information, and AI programs had no significant association (*p* > 0.05) with study sites. Anestrous (30.5%) and repeat breeders (38.9%) were among the causes of the low conception rate during the synchronization program. There were no significant variations in terms of AI program constraints in the three districts.

**Conclusion:**

AI technicians and farm owners need continuous training to improve their heat detection skills, increase their knowledge, and obtain a successful program.

## 1. Introduction

Ethiopia is ranked first in Africa, with an estimated 53.99 million heads of cattle. Of these breeds, local breeds are 98.95 percent of the total cattle in the country, and the rest are mixed and exotic breeds, comprising between 0.94 percent and 0.11 percent, respectively [1]. The total livestock population of the Tigray region is approximately 2,713,750 cattle, representing 7.0% of Ethiopia's total cattle. The population involves the majority of local breeds and some exotic crossbreeds of cattle on specified farms [1, 2]. In Ethiopia, milk production relies mostly on the genetic resources of indigenous livestock and the highest contribution is made by cattle (81.2%) [3]. In Ethiopia, dairy production is still substantial and indigenous cows have an estimated daily milk production of 1.37 liters per day [1, 3].

Synchronization of ovulation is the process by which the reproductive cycle of animals is manipulated by the use of hormones or their analogues to induce ovulation at a precise point in time [4, 5]. Depending on the protocol, the estrous cycle is regulated with one or more hormones to carry females into the estrus (heat) at a similar time to the producer's choice. The hormonal profile and functional structures present in the ovaries at various stages of the estrus cycle are critical for the selection and successful execution of programmed estrus synchronization [6]. The fundamental method is to control the timing of estrus initiation by regulating the estrous cycle duration. The various approaches to control cycle length are prostaglandin administration to regress the corpus luteum (CL) of the animal prior to natural luteolysis and the use of gonadotropin-releasing hormones (GnRH) and an analog to temporarily suppress ovarian activity or new ways to create estrous synchrony [7].

In recent years, Ethiopian governmental organizations (GOs) and NGOs have demanded estrus synchronization programs in various areas to place significant proportions of milk cattle on the river for brief, predetermined cycles and to improve AI productivity by improving the efficiency of estrus detection. On-farm estrus synchronization in different parts of the country has proven effective [4]. The region of Tigray was among those areas that introduced on-farm estrus synchronization, resulting in a medium design rate and some factors influencing the synchronization software. The present study was a cross-sectional study conducted to assess the efficacy of on-farm estrus synchronization in smallholder and private farms of Agula, Wukro, and Mekelle, Tigray, Northern Ethiopia.

## 2. Materials and Methods

### 2.1. Study Area

**Figure 1:**
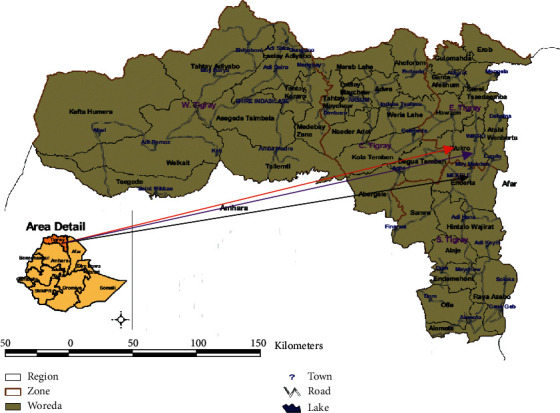
Map of the three study districts.

The study was carried out from November 2019 to February 2020 in three areas of the Tigray region: Agula, Wukro, and Mekelle. Mekelle is the district of Tigray, 783 kilometers north of Addis Ababa. It is located at 13°32′N and 39°33′E and has a human population of 215,546. It has an average altitude of 2200 meters above sea level, with mean minimum and maximum monthly temperatures of 8.7°C and 26.8°C, respectively. The annual average rainfall in Mekelle is 600 mm Hg, and more than 70% of it falls between July and August. The long dry season extends from October to May (Figure 1) [2].

Wukro town lies 45.6 km and is found along the Genfel River in the eastern zone of Tigray, with a population of 30,210 people. It is located at 13°47′N 39°36′E latitude and longitude with an elevation of 1,972 meters above sea level. The area has an average temperature of 22°C with 26% humidity. Agula lies approximately 32 km northeast of Mekelle and has a latitude and longitude of 13°41′30″N 39°35′30″E with an elevation of 1930 meters above sea level. The total livestock population of the Tigray region is approximately 2,713,750 cattle, representing 7.0% of Ethiopia's total cattle (Figure 1). The population involves the majority of local breeds and some exotic crossbreeds of cattle on specified farms (Table 1) [1].

### 2.2. Study Population and Design

The animals used in this research were cows of various breeds and ages using estrous synchronization and artificial insemination technologies. A total of 154 owners of dairy farms were interviewed for this report. The farm management scheme was almost identical, i.e., most of the animals were housed in the intensive system.

For the evaluation of factors affecting the performance of on-farm estrus synchronization, a cross-sectional questionnaire-based research design was used across milk farms in the study region. A semistructured questionnaire survey on different aspects of factors influencing the effectiveness of estrus synchronization collected the results.

### 2.3. Sampling Technique and Sample Size Determination

Based on the supply of dairy cows using synchronization and artificial insemination techniques, dairy farms were intentionally sampled. A purposive sampling technique was the sampling method used here. Dairy cow estrus synchronization knowledge, milk cattle production ability, and usability were taken into account before choosing representative samples. In the interview from the sample area, a total of 154 dairy farmers were deliberately chosen.

#### 2.3.1. Method of Data Collection

After rapid informal field research and consultation with AI technicians, farmers' organizations were chosen from three districts. During the participant selection process, certain criteria such as milk cattle development capacity, farm accessibility, expertise experience with milking cows, and estrus synchronization were considered. A total of 154 dairy farmers were interviewed using a semistructured questionnaire. The questionnaire has the following subtopics: demographics, farm practices, breeding management, and constraints.

### 2.4. Data Management and Statistical Analysis

Data were coded, entered, and managed by Microsoft Office Excel (2016) and then analyzed by using Stata release 13 statistical software. Descriptive analysis was used to describe the results of the proportion analysis. Chi-square (*X*^2^) tests for categorical variables were used to test the relationship between the dependent variable and different independent variables. The association of the different risk factors with farm estrus synchronization practice was calculated. A statistically significant association between variables was considered to exist if the computed *p* value was less than 0.05.

## 3. Results

### 3.1. General Household Characteristics of the Respondents

In the current study, a total of 154 respondents were interviewed to evaluate factors affecting the effectiveness of estrus synchronization in selected farm dairy cattle in Agula, Wukro, and Mekelle. Out of the total respondents, 73 (47.4%) were in the age range of 18–25 years, 26 (16.9%) were in the range of 26–35 years, 38 (24.7%) were in the age range of 36–45 years, and the remaining 17 (11.0%) were in the age range of greater than 45 years.

Regarding the educational background of the respondents, most participants were informally educated, which accounted for 61 (39.6%) of the total, followed by illiterate participants (47, 30.5%) and primary school participants (40, 25.9%). In the study area, female households were the most involved and active participants of the synchronization program (79, 51.3%), and male households and both males and females were almost equally involved, accounting for approximately 24.0%. The age and family members who followed up on AI and synchronization had significant variation (*p* < 0.05) among the study sites; however, the education level and marital status of the participants had no significant association (*p* > 0.05) within the different study sites (Table 2).

### 3.2. Farmers' Practice toward Farm Management Systems in Agula, Wukro, and Mekelle City

As indicated in Table 3, most participants in the studied areas kept mainly exotic (68, 44.2%) and crossbreed animals (61, 39.6%), respectively. Artificial insemination, which accounts for approximately 98%, is the major breeding technology commonly practiced in all study districts; however, the breeding practice has no significant association (*p* > 0.05) within these districts. Regarding their management system, most of the studied dairy farms commonly practice intensive farming (113, 73.4%), followed by semi-intensive (18.2%) and extensive farming (8.4%). In all the study districts, the majority of the studied farms were large-scale farms. Most farms feed their animals mainly a mixture of roughage and concentrate. Most farms provide feed and water at a frequency of two times per day. Factors such as breed, management system, feed type, feeding, and watering frequency had a significant association (*p* < 0.05) with the study sites (Table 3).

### 3.3. Knowledge and Attitude of Farmers regarding Estrus Synchronization and AI Programme at the Study Sites

According to the current study, 139 (90.3%) of the participants knew the concept of estrus synchronization, and they obtained this knowledge through an extension program provided to the owners by the government (57, 37.0%). From a farmer association, 31 (20.1%) and 27 (17.2%) farmers gained information from the radio. Since Ethiopia is a country with a culture of communication with neighbors, a total of 24 (15.6%) farmers obtained this information from their neighbors. Additionally, the participants had knowledge of AI acquired by training and from the community where they lived.

A total of 118 (76.6%) were satisfied with the synchronization program and AI service. Most farmers use AI services more than once, and their animals are conceived. Some failures occurred during AI due to the disease condition of the animal from the total of 53 (34.4%) and due to the low performance of the technician (14, 9.1%), and some of the failures were caused by both the above conditions from the total of 35 (22.7%). The cause of failure during AI, awareness about synchronization, satisfaction with the AI, and estrus synchronization service have a significant variation (*p* < 0.05) within the three districts. On the other hand, the cause of AI failure, awareness about AI programs, source of synchronization information, and AI programs had no significant association (*p* > 0.05) with study sites (Table 4).

### 3.4. Major Constraints of the AI Program in the Study Area

In the current research, the major constraints that the participants faced within the farm of the three districts were anestrous of the total (59, 38.8%) and repeat breeder (95, 61.7%). There were no significant variations in terms of AI program constraints in the three districts (Table 5).

According to Table 6, in the study area, the farmers reared local (25, 16.2%), exotic (68, 44.2%), and crossbreed (61, 39.6%) cattle, and their association with conception rate was statistically significant (*p* < 0.05). Additionally, the participants in the study area kept their animals in intensive (113, 73.4%), semi-intensive (28, 18.2%), and extensive (13, 8.4%) system, and the association of the management system with the conception rate was statistically significant (*p* < 0.001). The participants fed their animals with a feed type of roughage (28, 18.2%) and concentrate (26, 16.9%), and the association of feed type with conception rate was statistically significant (*p* < 0.001).

The research results showed that the farmers used AI for one year (11, 10.4%), for two years (50, 32.5%), and for three and more years (88, 57.1%), and there was a statistically significant (*p* < 0.001) association between experience of AI use and conception rates. Originally, crossbreed animals came from purchasing crossbreed bulls (3, 1.9%) and cows or heifers (87, 56.5%) from the office of agriculture and rural development (60, 38.9%) and crossbreed bulls from the surrounding (4, 2.6%), and the association of crossbreed animal origin with extension services was statistically significant (*p* ≤ 0.0001) (Table 6).

## 4. Discussion

In most developing countries, including Ethiopia, dairy cattle farming has become an emerging market field in recent years to boost the economy of the larger community. As part of the development and development of the economy, livestock production and intensification are becoming one of the focus areas of the country's governmental and private sectors. However, much needs to be achieved to develop overall dairy farm husbandry and management activities that are directly related to the knowledge and practical skills of the staff [8].

Despite results from numerous surveys in various parts of Ethiopia, most livestock owners in Ethiopia are illiterate [9, 10]. The current study finds that the majority of farmers are in informal education and secondary education, but there is no significant association between the breeding system and the level of education. Meanwhile, education improves psychological attitudes toward embracing innovative technologies, particularly information-intensive and management-intensive practices [11], which can be seen as a successful opportunity to exercise the enhancement of community-based breeds by estrus synchronization.

Estrus synchronization is a regular procedure to improve the useful reproductive life of livestock [12, 13]. In deciding on the implementation of new farming technology methods, education is very significant. While there is still no consensus on its implications for technological adoption, various studies have revealed the positive impact of education on farmers' acceptance of various agricultural technologies [14].

In a previous study, most farmers were interested in dairy cattle estrus synchronization technology at the beginning of the programme. However, the current study shows that a total of 152 (98.7%) respondents used extension services. Accessing knowledge through extension services decreases confusion regarding technical efficiency and hence can over time make the evaluation of a person from solely subjective to empirical easier to follow [15].

Previous research by Mizan [16] showed that 65.9% of participants responded that those who have no access to Holstein Friesian crossbreeding bulls are significantly higher (*p* ≤ 0.01) than those who have access (43.1%) either from fellows or their own farms (unknown genetic level), whereas the present study shows that farmers get crossbreed animals originally from the office of agriculture and rural development (60, 38.9%) and by purchasing crossbreed bull (3, 1.9%) and crossbreed cow or heifer (87, 56.5%) and crossbreed from the surrounding (4, 2.6%), and the association of crossbreed animal origin with extension service was found statistically significant (*p* ≤ 0.0001).

In the study by Mizan [16], approximately 53% of participants replied that they had a lack of awareness regarding the estrus synchronization technology of dairy cattle during the application of the program in the study area, whereas the current study revealed that 90.3% of farmers were aware of the concept of synchronization technology and they obtained this idea through extension agents, farmer associations, radios, and neighbors. The former study revealed that 47% of the participants engaged in the estrus synchronization programme of dairy cattle without knowing the technology.

According to the current study, 15 (9.7%) farmers had no concept of synchronization programs. Similarly, Tegegne et al. [5] reported that the involvement of farmers and experts in estrus synchronization was without knowledge of the programme. In the study by Mizan [16], farmers were questioned about their understanding of the technologies of dairy cattle hormone estrus synchronization, and 59.7% and 24.3% of those farmers had knowledge about estrus synchronization and received negative and positive feedback on the technology, respectively. The current study revealed that 118 (76.6%) of the farmers had positive feedback on estrous synchronization, whereas 36 (23.4%) of the farmers had negative feedback on estrous synchronization. Of those farmers who had negative feedback on the estrus synchronization programme, 7.8% of the animals did not conceive and/or had a low pregnancy rate. Additionally, this was due to conception failure during AI due to the disease condition of the animal (34.4%) and heat detection problems (33.8%).

Sen and Onder [17] determined the effect of estrus synchronization programmes on the parturition time and some reproductive characteristics of Saanen goats in Tukey's study and revealed that estrus synchronization shortened the length of the kidding period, concentrated the parturition time during daylight hours, and increased reproductive performance in Saanen goats. Furthermore, the findings revealed that estrus synchronization could minimize labor costs throughout the year by decreasing the kidding interval of the goat flock.

Similarly, according to Gizaw et al. [4], most farmers in various parts of Ethiopia included in the estrus synchronization scheme have low awareness/satisfaction. The same authors claimed that farmers are pleased by hormonal estrous technology, rather than by the rate of reaction to hormone therapy, based on the conception/pregnancy rates achieved.

According to Mizan [16], the AI distribution system in Ethiopia has been made ineffective for many causes. These include ineffectual administration, lack of incorporation of the AI service and feed packaging with livestock and feed, lack of adequate communication between stakeholders, weak insemination motivations and expertise, lack of user feedback such as liquid nitrogen, and the lack of adequate recording systems [18].

The present study shows that failure due to the low performance of technicians occurs in 14 (9.1%) of the total. Duncan et al. [19] stated that AI represents a small portion of Ethiopia's total reproduction processes, indicating the country's dysfunctionality of the AI system. Poor infrastructure, managerial and financial restrictions, poor heat detection, and productivity of AI technologists are some of the factors that lead to the failure of AI in the country [20].

In order of importance, the current study revealed that anestrous (38.8%) and repeat breeders (61.7%) were the major constraints faced by farmers in Agula, Mekelle, and Wukro. The study by Mizan [16] revealed that heat detection problems (36%), efficiency of AI technicians (29.3%), absence of AI technicians (23.9%), and distance of AI centers (10.3%) were reported as the major constraints for low performance of synchronization and AI programs.

Heifers exhibited estrus earlier than cows, which may be because of differences in their follicular development. Cows and heifers of *Bos indicus* and *Bos taurus* breeds differ in their pattern of follicular development [21]. The study by Chebel et al. [22] showed that cows inseminated following estrus detection or timed AI had a similar conception rate. As the number of AIs increased, the conception rate (CR) decreased. Multiparous cows had lower CR than primiparous cows, and CR is affected by heat stress prior to AI, parity, number of AI, and postparturient diseases such as occurrence of milk fever and the retained placenta.

In the study of Mizan, 2019, in the field-level hormonal estrus synchronization scheme, only 31.5% had hormone-treated cows/heifers supplemented with multiple feed-staff combinations. In the current study, farmers kept their animals in the feeding system of grazing with supplementation for a total of 111 (97.4%). In agreement with the current study, Anzar et al. [23] showed that nutrition had a significant effect on the conception (*p* < 0.01) and efficiency of artificial insemination.

The study by Holm et al. [24] revealed that estrus synchronization following PGF2 treatment resulted in a considerable reduction in days to the first insemination in the test group and, finally, days to calving. Moreover, the first AI conception rate after PGF2 treatment was improved in this experimental study. The technician's skill may have been influenced by the increasing number of inseminations conducted per day. Estrus observation can be influenced by synchronization since a higher number of heifers in estrus at any given moment would result in more sexual behavior, such as mounting herd mates, making it easier to detect estrus symptoms.

The present study shows that, in the study area, the farmers reared local (25, 16.2%), exotic (68, 44.2%), and crossbred (61, 39.6%) animals and their association with conception rate was statistically significant (*p* < 0.05). Compared to the study by Mizan [16], there was no significant difference (*p* > 0.05) in the first service conception rate among crossbred (50.2%) and indigenous (45.0%) cows and/or heifers, but in this study, a higher conception rate was observed in crossbred cattle than in native cattle. The study by Anzar et al. [23] revealed that there was significant variation (*p* < 0.05) in conception rate among different species and breeds, and a higher conception rate was recorded in buffaloes than in cattle. Moreover, the conception rate was significantly higher with the semen of crossbred and buffalo bulls (*p* < 0.05).

The present study results revealed that farmers use AI (92.1%) and the association with conception rates is statistically insignificant (*p* > 0.39). The absence of significant differences between the AI and conception rate could be due to inaccuracy of heat detection, time and season of insemination, and skills of the AI technician. The study by Anzar et al. [23] to identify the factors that affect the success of AI services under field conditions revealed that factors such as species, milk production, body condition score, lactation state, heat signs, and uterine tone showed a significant (*p* < 0.01) effect on conception rate. Animals with mucus flow from external genitalia, noticeable uterine tone, good body condition, and lactating cows also had a significant effect on conception rate (*p* < 0.05). Because of AI technicians, the conception rate also fluctuated significantly (*p* < 0.01). The housing system and the time interval between initial heat symptoms and AI, on the other hand, had no effect on the conception rate (*p* > 0.05).

## 5. Conclusion

The current questionnaire survey revealed that many factors influence the success of estrous synchronization and the perception of the farmers toward the technology was low. While it was observed in the analyzed farm estrus synchronization software to encourage the estrus response rate, the pregnancy rate was lower. The poor performance rate was due to AI's inaccessibility, lack of AI connection to some degree and improved bull service, the problem of heat detection, lack of technology, knowledge of farmers, and cattle management, particularly feeding. Farmers lose their faith in AI because of their low performance. Lack of trust in synchronized AI also forced farmers to heat their cows without breeding or to make use of local bulls that did not interest them. In conclusion, improved bull services should be accessible in parallel for dairy owners. In addition, community awareness of synchronization and AI was created and farmers' negative attitudes toward estrus synchronization were changed. AI technicians and farm owners need continuous training to improve their skills in heat detection and increase their knowledge and obtain a successful program.

## Figures and Tables

**Table 1:** Summary of the geolocation of the three study areas.

	Mekelle	Agula	Wukro
Mean altitude (meters above sea level)	2,200	1,930	1,972
Latitude and longitude	13°32′N 39°33′E	13°41′30″N 39°35′30″E	13°47′N 39°36′E

**Table 2:** General household characteristics of the respondents in the study area.

Variables		Wukro, *N* (%)	Agula, *N* (%)	Mekelle, *N* (%)	Total, *N* (%)	Chi-square (*X*^2^)	*p* value
Age	Between 18–25	25 (54.4)	20 (37.7)	28 (50.9)	73 (47.4)	13.54	0.035
	Between 26–35	8 (17.4)	10 (18.9)	8 (14.6)	26 (16.9)		
	Between 36–45	12 (20.1)	18 (33.9)	8 (14.6)	38 (24.7)		
	>45	1 (2.17)	5 (9.4)	11 (20.0)	17 (11.0)		
Education	Illiterate	10 (21.7)	15 (28.3)	22 (40.0)	47 (30.5)	12.49	0.052
	Informal education	15 (32.6)	25 (47.2)	21 (38.2)	61 (39.6)		
	Primary school	17 (36.9)	13 (24.5)	10 (18.2)	40 (25.9)		
	Secondary school	4 (8.7)	0 (0.0)	2 (3.6)	6 (3.9)		
Responsible family member for follow-up AI and synchronization	Females only/wife	36 (78.3)	16 (30.2)	27 (49.1)	79 (51.3)	41.27	≤0.0001
	Males only/husband	6 (13.04)	26 (49.1)	6 (10.9)	38 (24.7)		
	Both females and males	4 (8.7)	11 (20.8)	22 (40.0)	37 (24.0)		
Marital status	Single	13 (28.3)	17 (32.1)	17 (30.9)	57 (37.01)	3.93	0.416
	Married	33 (71.7)	34 (64.2)	34 (61.8)	101 (65.6)		
	Divorced	0 (0.00)	2 (3.8)	4 (7.3)	6 (3.9)		

**Table 3:** Farmers' practices regarding the farm management system in the study districts.

Variables		Wukro, *N* (%)	Agula, *N* (%)	Mekelle, *N* (%)	Total, *N* (%)	Chi-square (*X*^2^)	*p* value
Breed	Indigenous/local	3 (6.5)	2 (3.8)	20 (36.4)	25 (16.2)	51.16	≤0.0001
	Exotic	10 (21.7)	30 (56.6)	28 (50.9)	68 (44.2)		
	Crossbred	33 (71.7)	21 (39.6)	7 (12.7)	61 (39.6)		
Breeding practice	Natural mating	0 (0.0)	3 (5.7)	0 (0.0)	3 (1.9)	5.83	0.054
	AI	46 (100.0)	50 (94.3)	55 (100.0)	151 (98.1)		
Management system	Intensive	39 (84.8))	48 (90.6)	26 (47.3)	113 (73.4)	37.51	≤0.0001
	Semi-intensive	7 (15.2)	5 (9.4)	16 (29.1)	28 (18.2)		
	Extensive	0 (0.00)	0 (0.0)	13 (23.6)	13 (8.4)		
Feed type	Roughage	4 (8.7)	11 (20.8)	13 (23.6)	28 (18.2)	54.74	≤0.0001
	Concentrate	1 (2.2)	24 (45.3)	1 (1.8)	26 (16.9)		
	Both type	41 (89.1)	18 (33.9)	41 (74.5)	100 (64.9)		
Feeding frequency	Two times per day	45 (97.8)	8 (15.1)	35 (63.6)	88 (57.1)	70.30	≤0.0001
	Three times per day	1 (2.2)	45 (84.9)	20 (36.4)	66 (42.9)		
	Ad-libitum	0 (0.0)	0 (0.0)	0 (0.0)	0 (0.0)		
Watering frequency	Two times per day	46 (100.0)	31 (58.5)	44 (80.0)	121 (78.5)	27.39	≤0.0001
	Three times per day	0 (0.0)	19 (35.9)	11 (20.0)	30 (19.5)		
	Ad-libitum	0 (0.0)	3 (5.7)	0 (0.0)	3 (1.9)		

**Table 4:** Knowledge and attitude of farmers regarding synchronization and AI programs at the study sites.

Variables	Category	Wukro, *N* (%)	Agula, *N* (%)	Mekelle, *N* (%)	Total, *N* (%)	Chi-square (*X*^2^)	*p* value
Awareness about synchronization	Yes	46 (100.0)	43 (81.1)	50 (90.9)	139 (90.3)	10.01	0.007
	No	0 (0.0)	10 (18.9)	5 (9.1)	15 (9.7)		
If yes, source of information about synchronization	Extension agent	21 (45.7)	19 (35.9)	17 (30.9)	57 (37.0)	3.65	0.724
	Farmer association	8 (17.4)	10 (18.9)	13 (23.6)	31 (20.1)		
	Radio	11 (23.9)	6 (11.3)	10 (18.2)	27 (17.5)		
	Neighboring farmers	6 (13.0)	8 (15.1)	10 (18.2)	24 (15.6)		
Satisfaction with the AI and synchronization service	Yes	46 (100.0)	36 (67.9)	36 (65.5)	118 (76.6)	20.10	≤0.0001
	No	0 (0.0)	17 (32.1)	19 (34.5)	36 (23.4)		
Awareness about AI program	Yes	46 (100.0)	49 (92.5)	54 (98.2)	149 (96.8)	5.02	0.081
	No	0 (0.0)	4 (7.5)	1 (1.8)	5 (3.2)		
If yes, source of information about AI program	By training	28 (60.9)	31 (58.5)	35 (63.6)	94 (61.0)	0.54	0.765
	From community	18 (39.1)	18 (33.9)	24 (43.6)	60 (38.9)		
	No	0 (0.00)	2 (3.8)	10 (18.2)	12 (7.8)		
Failure during AI	Yes	33 (71.7)	26 (49.1)	43 (78.2)	102 (66.2)	11.13	0.004
	No	13 (28.3)	27 (50.9)	12 (21.8)	52 (33.8)		
If yes, cause of AI failure	Disease condition of the animal	19 (41.3)	11 (20.8)	23 (41.8)	53 (34.4)	1.89	0.756
	Heat detection problem	17 (36.9)	10 (18.9)	25 (45.5)	52 (33.8)		
	Low performance of the technician	5 (10.9)	4 (7.5)	5 (9.1)	14 (9.1)		

**Table 5:** The major constraints of the AI program in the study area.

Constraints of AI program	Wukro, *N* (%)	Agula, *N* (%)	Mekelle, *N* (%)	Total, *N* (%)	Chi-square (*X*^2^)	*p* value
Anestrous	17 (28.8)	24 (40.7)	18 (30.5)	59 (38.8)	1.85	0.396
Repeat breeder	29 (30.5)	29 (30.5)	37 (38.9)	95 (61.7)		

**Table 6:** Association of conception rate, extension service, and education level with different factors in the study area.

Variables	*X* ^2^	*p* value
Conception rate		
Breed	24.35	0.05
Breeding system	0.26	0.611
Farm type	0.81	0.668
Management system	42.54	0.001
Feed type	20.59	0.001
Feeding frequency	1.69	0.193
Number of AI services	4.71	0.194
Experience in AI use	14.70	0.001

Extension service		
District	0.86	0.649
Management system	0.74	0.692
Breeding system	0.04	0.841
AI frequency	1.21	0.751
Crossbreed animal origin	18.78	0.001

Education level		
Breeding system	2.25	0.521
Management system	8.10	0.231
Concept of synchronization	1.37	0.714
Concept of AI	3.86	0.277
Animals conceived after service	2.22	0.527
Experience in AI use	6.26	0.395

## Data Availability

The data analyzed during this study will be provided on request from the corresponding author.

## Abbreviations

AI: Artificial insemination
CL: Corpus luteum
CR: Conception rate.
